# Overexpression of the Wild Soybean Expansin Gene *GsEXPB1* Enhances Salt Stress Tolerance in Transgenic Soybeans

**DOI:** 10.3390/plants14182851

**Published:** 2025-09-12

**Authors:** Linlin Wang, Yanlin Sun, Wenxu Liu, Xiaolei Shi, Jing Ma, Fumeng He, Fenglan Li, Xu Feng

**Affiliations:** 1 College of Life Sciences, Northeast Agricultural University, Harbin 150030, China; 18315407194@163.com (L.W.); 18366711229@163.com (Y.S.); 18845490588@139.com (W.L.); 15184523620@163.com (J.M.); hefumeng@neau.edu.cn (F.H.); 2 Crop Research Institute, Xinjiang Uygur Autonomous Region Academy of Agricultural Sciences, Urumqi 831399, China; xl19961125@163.com; 3 Heilongjiang Green Food Science Research Institute, Harbin 150023, China; 4 Heilongjiang Provincial Engineering Technology Research Center for High-Quality Agricultural Functional Fungi Agents, Harbin 150030, China

**Keywords:** wild soybean, expansin gene *GsEXPB1*, salt stress tolerance, soybean, hairy root

## Abstract

As the ancestor and close relative of soybeans, wild soybeans exhibit strong salt tolerance and are ideal materials for discovering salt-tolerant genes. Expansins are a type of cell wall-loosening protein that plays an active role in regulating plant salt tolerance. We previously obtained the wild soybean expansin gene *GsEXPB1*, which is specifically transcribed in roots and actively responds to salt stress. Overexpression of this gene significantly promotes the growth of soybean hairy roots under salt stress. To further elucidate the function of the gene in regulating plant tolerance to salt stress, this study obtained soybean hairy roots that overexpress the *GsEXPB1* gene and silence its homologous gene *GmEXPB4* through RNAi. Under salt stress, the overexpression of the *GsEXPB1* gene significantly promoted the growth of soybean hairy roots, while the hairy roots that were silenced for the *GmEXPB4* gene exhibited an opposite phenotype. Physiological assay results indicate that *GsEXPB1* enhances the tolerance of soybean hairy roots to salt stress by regulating the antioxidant system and Na^+^/K^+^ content. In soybean lines overexpressing *GsEXPB1*, the germination rate of seeds and root growth indicators under salt stress were significantly improved compared to those of wild-type plants. Meanwhile, *GsEXPB1* enhances the tolerance of transgenic lines to salt stress by actively regulating the antioxidant system, osmotic adjustment system, chlorophyll content, cell wall components, and Na^+^/K^+^ levels, significantly promoting growth and increasing the number of flowers and grain weight. This study reveals the physiological mechanism by which *GsEXPB1* enhances soybean salt tolerance, providing a theoretical basis and relevant references for the application of this gene in the breeding of new soybean salt-tolerant varieties.

## 1. Introduction

Soybean (*Glycine max*) is a major economic crop globally, providing abundant protein and oil. Its unique nitrogen-fixing ability makes it highly profitable in crop rotation systems and intercropping cultivation modes [1]. However, over long-term evolutionary selection and breeding domestication, the genetic diversity of its population has continuously decreased, with a significant loss of high-quality genes closely related to environmental adaptability. This has led to a continuous decline in its resistance to abiotic stresses, particularly its tolerance to salt stress [2]. Salt stress is a severe abiotic stress that significantly affects multiple critical stages of soybean growth [3], leading to a substantial decrease in soybean quality and yield [4]. Studies have shown that approximately 1 billion hectares of land worldwide are affected by salinization [5], and the yield loss caused by salt stress accounts for about 20.0% of global yield loss [6], which has already had a certain impact on the global food supply system [7]. Due to the aforementioned factors, there is an urgent need for untapped genetic resources from wild relatives of soybeans to enhance the salt tolerance of soybeans. Wild soybean (*Glycine soja*), as a wild relative of soybeans, has developed unique environmental adaptability through long-term natural selection and possesses excellent resistance genes [8]. Wild soybeans retain higher genetic diversity and adaptability to harsh environments [9]. Under salt stress, wild soybeans exhibit significant advantages in maintaining the stability of photosynthesis, with a lower inhibition of photosynthesis, and reduce the damage caused by salt stress by maintaining the balance of ion transport systems and improved levels of amino acid, carbohydrate, and polyol metabolism [10]. The water channel protein TIP2, isolated from wild soybeans and exhibiting response characteristics to salt stress, plays a significant role in enhancing the tolerance of *Saccharomyces cerevisiae* and soybean hairy roots to salt stress [11]. Feng et al. obtained a protein kinase, GsSnRK1, that responds to salt stress in wild soybeans; identified its phosphorylated substrate transcription factor, GsERF7; and revealed a new pathway through which GsSnRK1 cooperatively regulates GsERF7 to enhance soybean salt and alkali tolerance [12]. Exploring high-quality salt-tolerant gene resources from wild soybeans and applying them to the breeding of new soybean varieties holds significant value in enhancing the tolerance of soybean to salt stress and enriching genetic diversity [13,14].

Expansins were first discovered in the hypocotyls of cucumber (*Cucumis sativus*) in 1992. They loosen plant cell walls with non-enzymatic activity and are pH-dependent [15]. Members of the expansin family originate from a common ancestor and evolved into four subfamilies: α-expansins (EXPAs), β-expansins (EXPBs), expansin-like A (EXLA), and expansin-like B (EXLB) [16]. This family protein is relatively conserved in evolution, with amino acid homology among subfamilies typically ranging from 20% to 25%. It usually possesses two conserved domains: DPPB_1 (catalytic region) and Pollen_allerg_1 (binding region) [17]. Expansins, acting as cell wall-loosening proteins, play a crucial role in the plasticity of plant cell walls [18]. Numerous studies have demonstrated that expansins are involved in key regulatory roles in various growth processes, including seed germination [19], root development [20], leaf and stem growth [21,22], stomatal opening and closing [23], flower development [24], and fruit ripening [25]. Since plant cells interact with their environment through the mediation of the cell wall, expansins are directly involved in the plant’s response to environmental stress [26].

Expansins play a positive role in enhancing plants’ resistance to salt stress. Under high-salt conditions, plants are generally subjected to stress in three ways: oxidative stress, osmotic stress, and ionic stress. Low water potential disrupts the steady state of Na^+^/K^+^ ions in the cytoplasm, inducing toxic ion effects and subsequently causing oxidative damage [27]. However, expansins are effective in alleviating these damages in plants. The expansin ZmEXPBs from *Zea mays* can enhance the extensibility of the cell wall under salt stress, thereby better maintaining the integrity of the cell structure [28]. Under salt stress, expression of the expansin gene *SmEXPA13* from *Salix matsudana* is induced, and tobacco (*Nicotiana tabacum*) lines transformed with this gene effectively balance the Na^+^/K^+^ levels compared to wild-type plants under salt treatment [29]. Similarly, overexpressing the expansin gene *TaEXPA2* from wheat (*Triticum aestivum*) in tobacco results in transgenic plants with improved Na^+^/K^+^ regulation and antioxidant capacity, thereby enhancing their tolerance to salt stress [30]. The RNAi lines of the tobacco expansin gene *NtEXPA4* exhibit increased sensitivity to salt stress, while the overexpressing plants show the opposite phenotype, including the accumulation of osmoregulatory substances such as soluble sugars and proline (Pro) [31].

Although there have been numerous studies on the salt tolerance of wild soybean, there are relatively few reports focusing on expansin. The whole-genome identification of the soybean expansin family was completed by Zhu et al. (2014), with a total of 75 expansin family members identified [32]. Our team conducted a systematic identification of the expansin family members in wild soybeans in the early stage and carried out gene function research. There are a total of 75 expansin genes in wild soybeans. Combining the basic transcriptome data of nutrient organs, we obtained some expansin genes that are specifically expressed in the roots and actively respond to salt stress transcription, such as *GsEXLB14*, *GsEXPA36*, *GsEXPB1*, *GsEXLB2*, and *GsEXLB4*. Further research has found that overexpressing the expansin gene *GsEXLB14* can enhance the tolerance of soybean hairy roots to salt stress, and the transcription of genes encoding peroxidase, H^+^ transport ATPase, anion channel proteins, etc. is significantly upregulated in transgenic hairy roots [33]. Research data on GsEXPB1 reveal that this protein is localized to the cell wall. Overexpression of *GsEXPB1* significantly promotes the growth of soybean hairy roots and enhances their tolerance to salt stress, demonstrating the potential positive effect of *GsEXPB1* on improving soybean resistance to salt stress [34]. To further elucidate the functional mechanism of *GsEXPB1*, this study utilized transgenic soybean hairy roots and soybean lines as materials to analyze the function and physiological mechanism of *GsEXPB1* in enhancing the tolerance of soybean to salt stress, aiming to provide a reference for the application of this expansin gene in the salt-tolerant breeding of cultivated soybean.

## 2. Results

### 2.1. Phenotype Observation of Soybean Hairy Roots Overexpressing the GsEXPB1 Gene and Silenced by RNAi for the GmEXPB4 Gene

**Figure 1:**
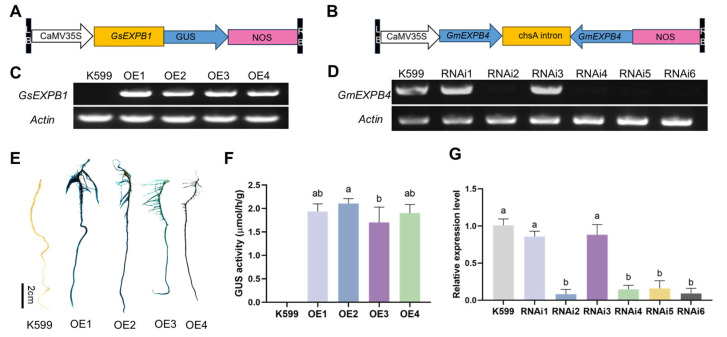
Construction of expression vectors and detection of transgenic soybean hairy roots. Note: (**A**) Schematic diagram of the overexpression vector pBI121-GsEXPB1-GUS construction; (**B**) schematic diagram of the RNAi-silencing vector pFGC5941-GmEXPB4 construction; (**C**) RT-PCR detection results of soybean hairy roots overexpressing the *GsEXPB1* gene; (**D**) RT-PCR detection results of soybean hairy roots with RNAi-silenced *GmEXPB4* gene; (**E**) GUS staining results of soybean hairy roots overexpressing the *GsEXPB1* gene; (**F**) GUS activity assay results of soybean hairy roots overexpressing the *GsEXPB1* gene; (**G**) qRT-PCR results of soybean hairy roots with RNAi-silenced *GmEXPB4* gene. Different lowercase letters indicate significant differences between different soybean hairy roots. *p* < 0.05.

**Figure 2:**
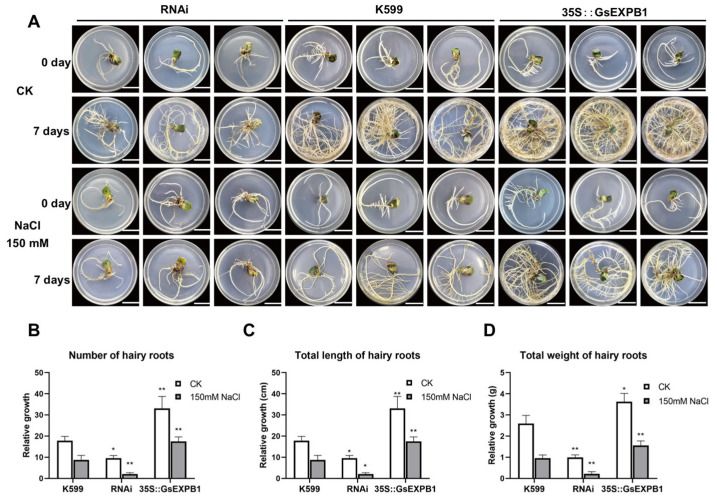
Phenotypic observation and quantitative results of transgenic soybean hairy roots. Note: (**A**) Phenotype of transgenic soybean hairy roots under normal and salt stress conditions. The K599 group served as the control group for hairy roots induced by empty bacteria, while the RNAi group was the *GmEXPB4* gene silencing group (bar = 2 cm). (**B**) The relative increase in root hair number. (**C**) The relative increase in total length of hairy roots. (**D**) The relative increase in total weight of hairy roots. “*” indicates that the phenotypic quantification data of transgenic soybean hairy roots showed significant differences compared to the K599 control group under the same cultivation conditions. * *p* < 0.05, ** *p* < 0.01; *n* = 12.

To further characterize the function of *GsEXPB1*, hairy root transformation experiments were used to obtain soybean hairy roots that overexpress the *GsEXPB1* gene and silence the *GmEXPB4* gene via RNAi. Schematic diagrams of the construction of expression vectors used for overexpressing the *GsEXPB1* gene and silencing the *GmEXPB4* gene via RNAi are shown in Figure 1A,B, respectively. Results from RT-PCR (Figure 1C) and the GUS staining and activity assay (Figure 1E,F) indicated successful expression of the *GsEXPB1* gene in soybean hairy roots, with a transformation efficiency of 83% (*n* = 100). The results of RT-PCR (Figure 1D) and qRT-PCR (Figure 1G) indicate that the *GmEXPB4* gene was successfully silenced in soybean hairy roots, with a silencing efficiency of 68% (*n* = 100). The phenotype analysis results of hairy roots are presented in Figure 2. Under normal culture conditions, the growth status of soybean hairy roots overexpressing the *GsEXPB1* gene was significantly better than that of the K599 group, while the growth status of soybean hairy roots silenced by RNAi for the *GmEXPB4* gene was weaker. After treatment with 150 mM NaCl stress, the growth of soybean hairy roots in all groups was significantly affected. However, the soybean hairy roots overexpressing the *GsEXPB1* gene showed a better state compared to the other two groups, while the RNAi group barely grew. Phenotype quantification results showed that under salt stress, the relative increase in the number of hairy roots in the overexpressing group was 99% higher than that in the K599 group, and the relative increases in total root length and root weight were 109 and 63%, respectively, demonstrating a significant growth-promoting effect.

### 2.2. Detection of Physiological Indexes of Transgenic Hairy Roots Under Salt Stress

**Figure 3:**
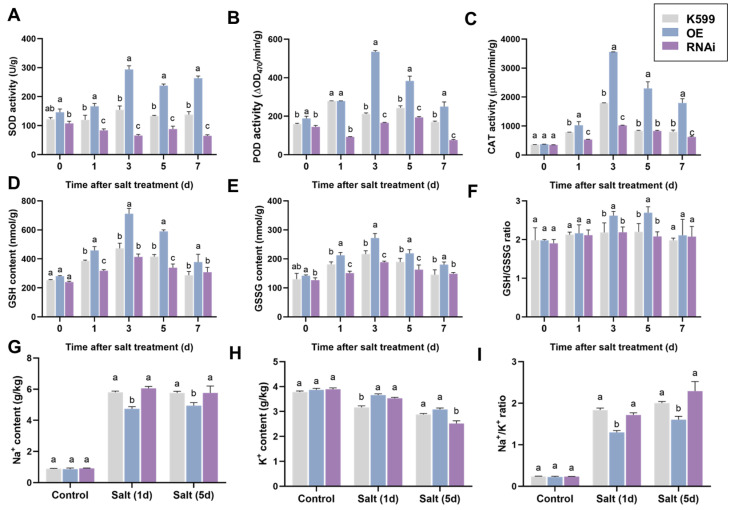
The detection results of physiological indices in transgenic soybean hairy roots under salt stress. Note: (**A**) The activity of SOD enzyme; (**B**) the activity of POD enzyme; (**C**) the activity of CAT enzyme; (**D**) the content of GSH; (**E**) the content of GSSG; (**F**) the ratio of GSH/GSSG; (**G**) the content of Na^+^ ions; (**H**) the content of K^+^ ions; (**I**) the ratio of Na^+^/K^+^. The K599 served as the control group for hairy roots induced by empty bacteria. The OE represented the group overexpressing the *GsEXPB1* gene, while the RNAi represented the group silencing the *GmEXPB4* gene. Indicated by different lowercase letters, there are significant differences among different soybean hairy roots at the same treatment time point. *p* < 0.05; *n* = 9.

We measured the antioxidant capacity-related indicators and the contents of Na^+^ and K^+^ in soybean hairy roots overexpressing the *GsEXPB1* gene and silencing the *GmEXPB4* gene by RNAi (Figure 3). The results showed that after treatment with 150 mM NaCl, the activities of antioxidant enzymes (SOD, POD, and CAT) and the contents of antioxidant substances (GSH and GSSG) in both the control group and transgenic soybean hairy roots exhibited an upward trend. However, compared to the control group, overexpressing the *GsEXPB1* gene significantly enhanced the antioxidant enzyme activities and the accumulation of antioxidant substances in soybean hairy roots, while the roots silencing the *GmEXPB4* gene by RNAi showed the opposite result. Three days after stress treatment, the activity of the SOD enzyme in OE hairy roots increased by 89.72% compared to K599, while it decreased by 57.41% in RNAi hairy roots (Figure 3A). The contents of GSH and GSSG in OE hairy roots increased by 50.83 and 25.51%, respectively, compared to the control group, while the related contents in RNAi hairy roots were lower than those in the control group (Figure 3D,E). The calculated GSH/GSSG ratio showed no significant difference between the RNAi group and the OE group during the treatment period, while that of the OE group significantly increased on days 3 and 5 after treatment (Figure 3F).

We also measured the Na^+^ and K^+^ contents in hairy roots under salt stress (Figure 3G,H), finding that, compared to the control treatment, the Na^+^ content and Na^+^/K^+^ ratio in the hairy roots of the K599 group, OE group, and RNAi group all increased, while the K^+^ content slightly decreased. Under control conditions, there were no significant differences in the Na^+^ and K^+^ contents among the hairy roots of each group. However, under salt stress, the Na^+^ content in the OE group decreased significantly compared to the K599 group, while the K^+^ content increased. The Na^+^ content in the RNAi group showed no significant difference from the K599 group, and although the K^+^ content increased significantly on day 1 after treatment, it decreased significantly on day 5 after treatment. It was found through calculation that the OE group maintained a lower Na^+^/K^+^ ratio compared to the K599 group, with a decrease of 29.32% on day 1 after treatment, while there was no significant change in the RNAi group.

### 2.3. Construction of a Soybean Strain Overexpressing the GsEXPB1 Gene

**Figure 4:**
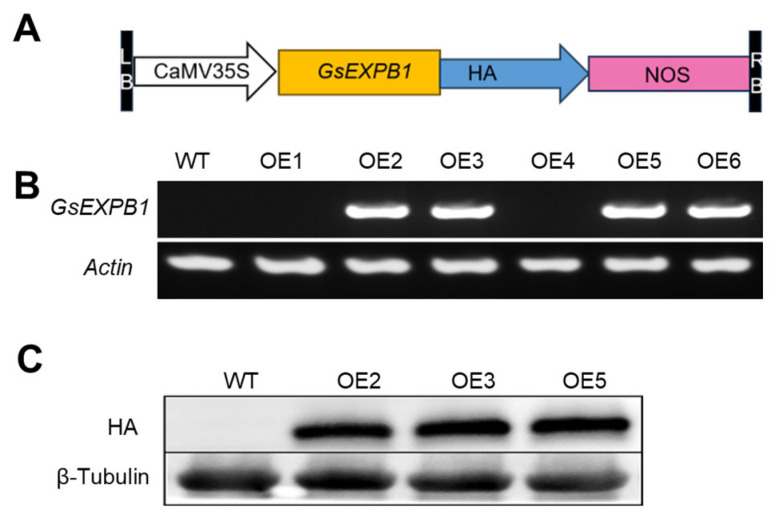
Construction of the overexpression vector for *GsEXPB1* gene and characterization of transgenic soybean lines. Note: (**A**) Schematic diagram of the structure of expression vector pTF101-GsEXPB1-HA; (**B**) RT-PCR identification results of transgenic soybean lines; (**C**) Western-blot detection results of transgenic soybean lines.

To further characterize the function of *GsEXPB1*, we obtained soybean lines overexpressing the *GsEXPB1* gene driven by the 35S promoter. RT-PCR (Figure 4B) and Western blot (Figure 4C) experiments indicated that this gene had been successfully expressed in soybeans. Based on the identification results, we selected three transgenic soybean lines, OE2, OE3, and OE5, for further experiments.

### 2.4. Analysis Results of Salt Tolerance of Transgenic Soybean Seeds

**Figure 5:**
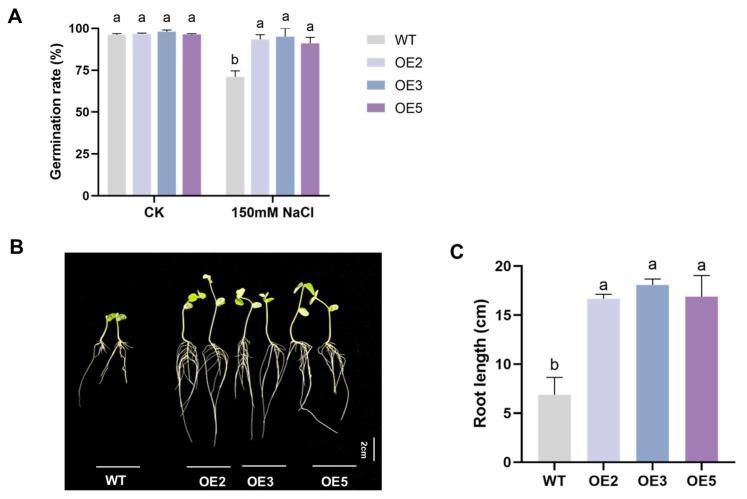
Analysis of salt tolerance in soybean seeds overexpressing the *GsEXPB1* gene. Note: (**A**) Seed germination rate, *n* = 100; (**B**) the growth status of soybean seedlings in a seed germination bag under salt stress; (**C**) root length of soybean seedlings in a germination bag under salt stress, *n* = 10. Different lowercase letters indicate significant differences between different soybean lines. *p* < 0.05.

We assessed the germination rate of transgenic soybean seeds under 150 mM NaCl treatment. The results indicated that under control culture conditions, there was no significant difference in this rate between transgenic soybean seeds and wild-type seeds. However, under salt stress treatment, the germination rate of transgenic seeds was significantly higher than that of wild-type seeds, both reaching over 90%. Specifically, the germination rates of seeds in the OE2, OE3, and OE5 groups were increased by 22.33, 24, and 20%, respectively, compared to the wild type (Figure 5A). The growth status of soybean seedling roots under 150 mM NaCl treatment was observed using seed germination bags (Figure 5B,C). The results showed that the growth of transgenic soybean roots was significantly better than that of the wild type, with the root lengths of OE2, OE3, and OE5 seedlings increasing by 142.48, 162.85, and 145.39%, respectively, compared to the wild type.

### 2.5. Analysis of Salt Tolerance of Transgenic Soybean Plants in the Seedling Stage

**Figure 6:**
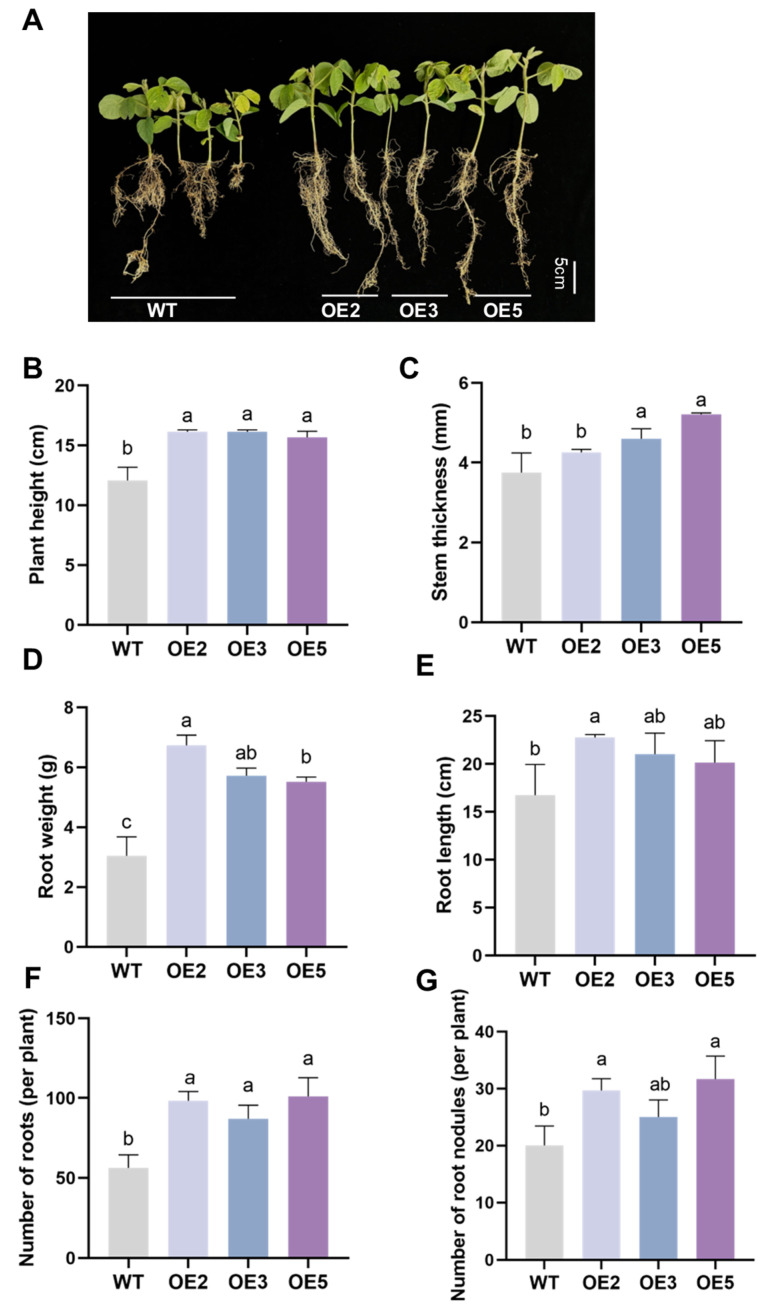
Phenotypic observation results of transgenic soybean seedlings under saline soil cultivation conditions. Note: (**A**) The growth stage of soybean seedlings; (**B**) plant height; (**C**) stem thickness; (**D**) root weight; (**E**) root length; (**F**) number of roots per plant; (**G**) number of root nodules per plant. Different lowercase letters indicate significant differences between different soybean lines. *p* < 0.05; *n* = 10.

**Figure 7:**
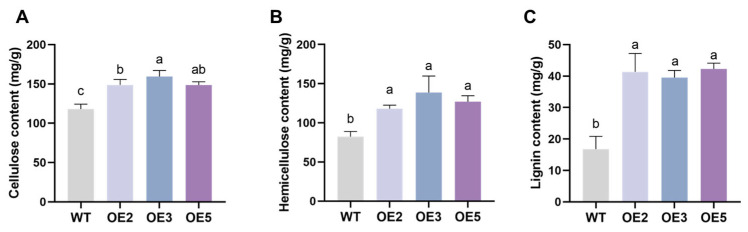
The content of cell wall components in the roots of transgenic soybean seedlings under saline soil cultivation conditions. Note: (**A**) Cellulose content; (**B**) hemicellulose content; (**C**) lignin content. Different lowercase letters indicate significant differences between different soybean lines. *p* < 0.05; *n* = 8.

We cultivated soybeans outdoors using naturally salinized soil to simulate field conditions. Observations of the seedlings on the 35th day after sowing showed that the growth status of the overexpressing lines was significantly better than that of the wild type (Figure 6A). Phenotype quantification results indicate that the overexpressing plants exhibited notable improvements in plant height, stem diameter, root weight, root length, number of roots per plant, and nodule count compared to the wild type. We also focused on the effect of *GsEXPB1*, as an expansin, on the content of cell wall components in transgenic soybean lines. Compared to the wild type, the overexpression of the *GsEXPB1* gene significantly affected the content of cell wall components in soybean roots, with a notable increase in the accumulation of cellulose, hemicellulose, and lignin (Figure 7). Specifically, compared to the wild type, the cellulose content in OE3 roots increased by 35.08%, and the hemicellulose content increased by 68.46%. The lignin content in OE2 roots increased by 146.16% compared to the wild type. These data suggest that the *GsEXPB1* gene may participate in cell wall construction and root growth by regulating the content of cell wall-related components.

**Figure 8:**
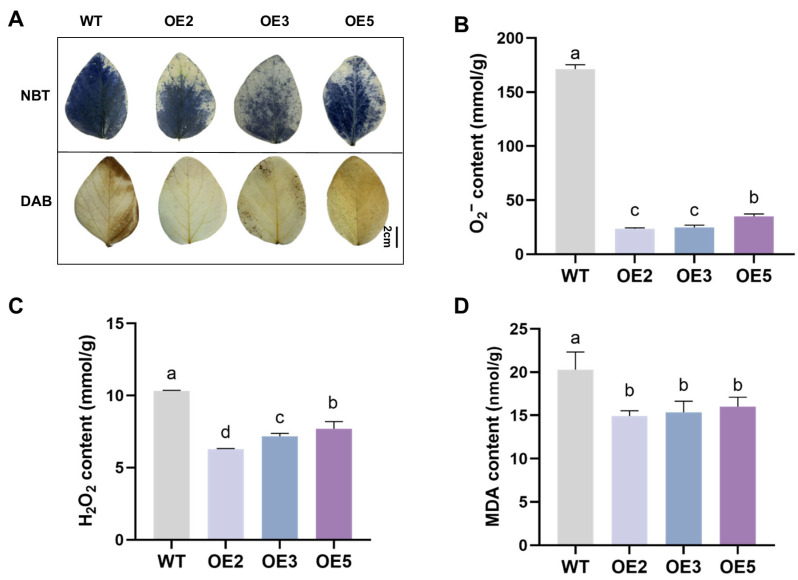
Results of reactive oxygen species staining and content determination of transgenic soybean seedlings under saline soil cultivation conditions. Note: (**A**) NBT and DAB staining results; (**B**) O_2_^−^ content; (**C**) H_2_O_2_ content; (**D**) MDA content. Different lowercase letters indicate significant differences between different soybean lines. *p* < 0.05; *n* = 8.

**Figure 9:**
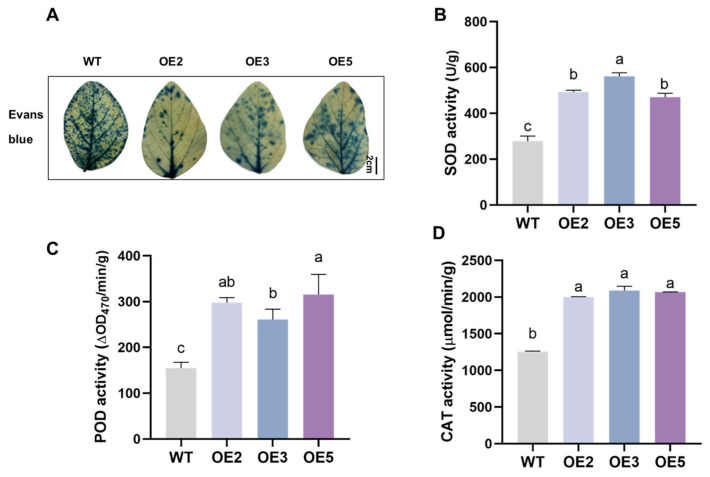
Evans blue staining and antioxidant enzyme activity determination results of transgenic soybean seedlings under saline soil cultivation conditions. Note: (**A**) Evans blue staining results; (**B**) SOD activity; (**C**) POD activity; (**D**) CAT activity. Different lowercase letters indicate significant differences between different soybean lines. *p* < 0.05; *n* = 8.

**Figure 10:**
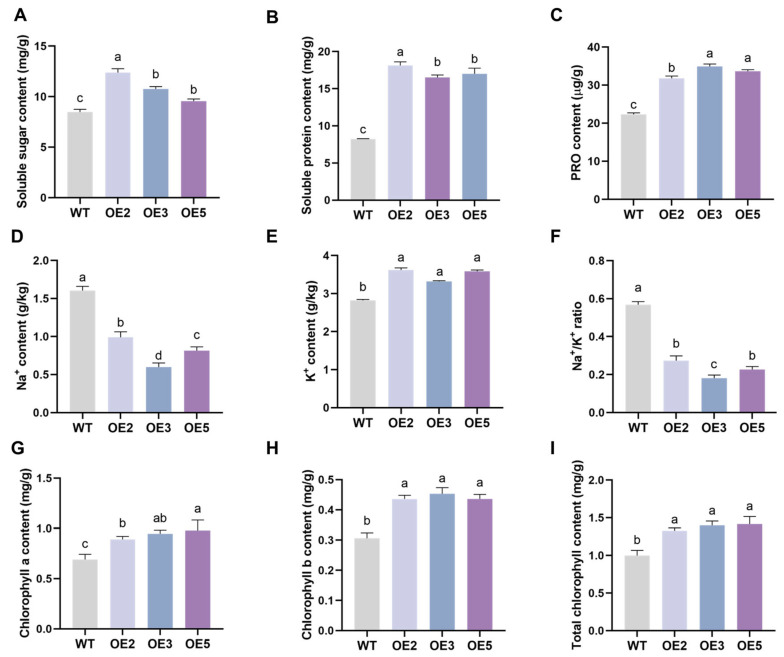
The determination results of osmotic adjustment substances, Na^+^ and K^+^ content, and chlorophyll content of transgenic soybean seedlings under saline soil cultivation conditions. Note: (**A**) Soluble sugar content; (**B**) soluble protein content; (**C**) proline content; (**D**) sodium ion content; (**E**) potassium ion content; (**F**) the ratio of sodium to potassium ion content; (**G**) chlorophyll a content; (**H**) chlorophyll b content; (**I**) total chlorophyll content. Different lowercase letters indicate significant differences between different soybean lines. *p* < 0.05; *n* = 8.

The NBT and DAB staining results (Figure 8A) showed that the leaves of the WT group plants were darker compared to those of the transgenic group, indicating a higher level of reactive oxygen species (ROS) accumulation. The results of O_2_^−^, H_2_O_2_, and MDA content measurements were consistent with the staining results (Figure 8B–D), indicating that the overexpression of *GsEXPB1* could significantly reduce the accumulation of ROS in transgenic soybean leaves. The Evans blue staining results (Figure 9A) showed that the degree of membrane damage in the cells of WT plant leaves was higher. Meanwhile, the activity monitoring results of three conventional reactive oxygen scavenging enzymes (SOD, POD, and CAT) indicated that the activity of the reactive oxygen scavenging enzyme system in the leaves of the overexpressing lines was stronger, significantly higher than that in the WT group (Figure 9B–D). The results from determining the osmotic adjustment substance content (Figure 10A–C) showed that overexpressing *GsEXPB1* promoted the accumulation of soluble sugar, soluble protein, and proline content in soybean plant leaves. Compared with the WT group, the soluble sugar content in the OE2 group increased by 45.95%, the soluble protein content increased by 120.38%, and the proline content increased by 42.41%, all showing significant differences.

The results of Na^+^ and K^+^ content determination in plant roots (Figure 10D–F) showed that the overexpressing group maintained lower Na^+^ and higher K^+^ levels, with a significantly lower Na^+^/K^+^ ratio compared to the wild type. Among them, the Na^+^/K^+^ ratio in OE3 decreased by 68.20% relative to the wild type, which was the most pronounced. In addition, we also measured the chlorophyll content in soybean leaves (Figure 10G–I), and the results indicated that the accumulation of chlorophyll content in the leaves of the overexpressing group plants was significantly higher than that for the wild-type group. Specifically, the total chlorophyll content in OE-5 increased by 41.74% compared to the WT group.

The measured data of the aforementioned physiological indicators indicate that overexpression of *GsEXPB1* can enhance the tolerance of transgenic soybean plants to salt stress by maintaining high antioxidant enzyme activity, osmotic adjustment substance content, chlorophyll level, and Na^+^/K^+^ content regulation ability and reducing the accumulation of reactive oxygen species. Thus, a better growth phenotype can be achieved in saline soil.

### 2.6. Phenotype Observation of Transgenic Soybeans During Flowering Period

**Figure 11:**
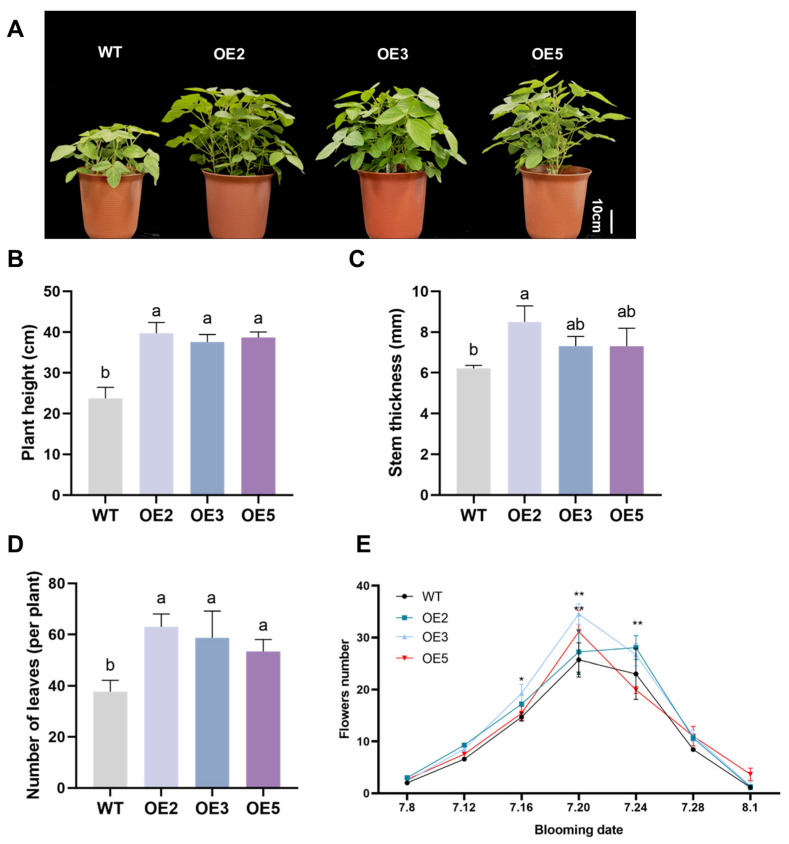
Phenotypic observation results of transgenic soybeans at the flowering stage under saline soil cultivation conditions. Note: (**A**) Phenotype observation of transgenic soybean plants; (**B**) plant height; (**C**) stem thickness; (**D**) number of leaves per plant; (**E**) flowering counts of soybean plants during the flowering stage. Different lowercase letters indicate significant differences between different soybean lines. *p* < 0.05; *n* = 10. “*” indicates that on the same day, there is a significant difference in flowering quantity between transgenic lines and wild type. * *p* < 0.05, ** *p* < 0.01; *n* = 10.

During the flowering stage, the phenotype of transgenic soybeans exhibited significant differences from that of wild-type soybeans (Figure 11A). Quantitative phenotypic data revealed that overexpression of the *GsEXPB1* gene significantly promoted the growth of soybean plants under cultivation conditions in saline soil. The plant height, stem diameter, and leaf count of transgenic soybeans were significantly higher than those of wild-type plants (Figure 11B–D). The statistical results of flowering quantity showed that the number of flowers was highest during the 12th to 16th day of the flowering period (20–24 July), and the number of flowers in the overexpressed lines was significantly higher than that in the wild type. On the 12th day of this period, compared with the wild type, the flowering quantity of OE3 and OE5 increased by 34.37 and 21.14%, respectively, indicating that the overexpression of *GsEXPB1* has a significant promoting effect on soybean flowering.

### 2.7. Observation of Mature Transgenic Soybean Phenotype

**Figure 12:**
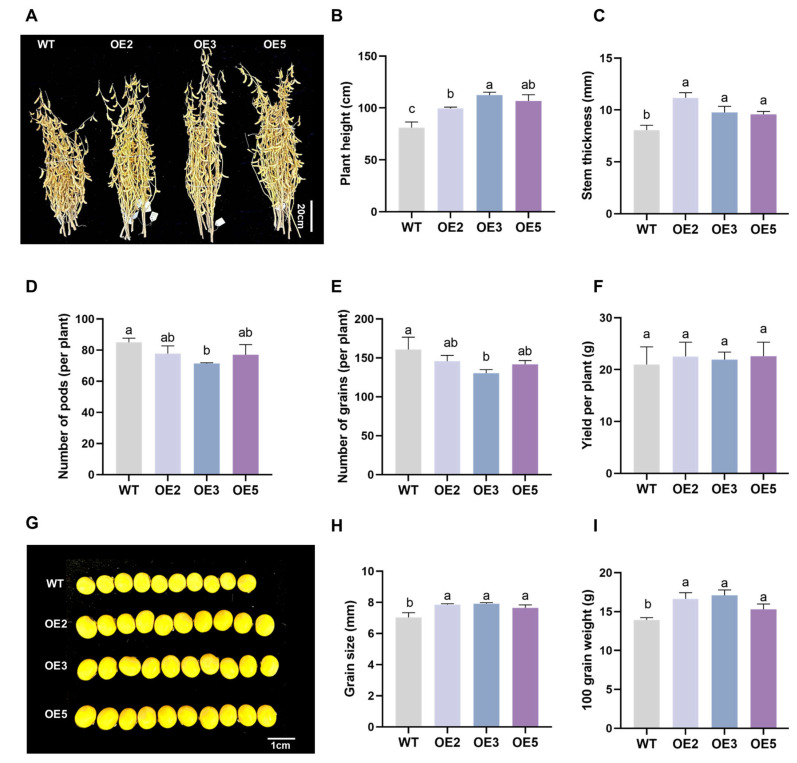
The phenotypic observation results of transgenic soybean plants at the maturity stage under saline soil cultivation conditions. Note: (**A**) The phenotype of mature soybean plants; (**B**) plant height; (**C**) stem thickness; (**D**) number of pods per plant; (**E**) number of grains per plant; (**F**) yield per plant; (**G**) phenotype of soybean seeds; (**H**) grain size; (**I**) weight per hundred grains. Different lowercase letters indicate significant differences between different soybean lines. *p* < 0.05; *n* = 8.

We observed and measured the phenotype and yield-related indicators of transgenic soybeans at maturity. At maturity, the plant height and stem diameter of transgenic soybean lines were significantly greater than those of the wild type. Specifically, compared to the wild type, the plant height of OE3 increased by 38.76%, and the stem diameter of OE2 increased by 38.65% (Figure 12A–C). To further investigate the impact of overexpressing the *GsEXPB1* gene on soybean yield during maturity, we conducted statistical analysis on the number of pods per plant, the number of seeds per plant, and the yield per plant (Figure 12D–F). The results showed that there were no significant differences in the number of pods per plant and the number of seeds per plant between OE2 and OE5 and the wild type, but OE3 was significantly reduced compared to the wild type. There was no significant difference in yield per plant between the transgenic lines and the wild type, but the overexpression of *GsEXPB1* significantly increased the seed size of soybeans, thereby significantly improving the 100-seed weight. Specifically, the seed size of OE3 increased by 12.26% compared to the wild type, and the 100-seed weight increased by 22.79% (Figure 12G–I).

## 3. Discussion

Plant growth typically involves two main pathways: the increase in cell number and the expansion of cell volume. The function of expansin has been proven to play a crucial role in the latter pathway. Furthermore, the cell wall serves as the first line of defense for plants against external environmental stress. The dynamic adjustment of their structure or composition is an important mechanism for plants to rapidly adapt to external stress. In turn, this adjustment determines the ability of expansin to regulate plant resistance to environmental stress. Our team’s previous research found through promoter prediction analysis that the transcription of wild soybean expansin is often closely related to plant growth and abiotic stress response [33]. Numerous studies have shown that expansin often plays a positive role in the above two aspects through effects such as promoting the growth of plant roots and enhancing resistance to environmental stress [34].

Wild soybean, the ancestor of cultivated soybean, possesses a high level of genetic diversity and abundant salt-tolerant gene resources, which holds significant breeding value for improving the salt stress tolerance of soybean [35]. Despite extensive research on wild soybean’s salt stress tolerance, reports focusing on the specific target of expansin are relatively scarce. We previously completed the whole-genome identification of wild soybean expansin and determined the basic transcriptome of wild soybean root, stem, and leaf organs. We found that the expansin gene *GsEXPB1* is specifically transcribed in the roots, and its transcript positively responds to increased salt stress. However, the function and mechanism of this gene in enhancing the tolerance of soybean to salt stress have not been clearly elucidated. Therefore, this study further investigated the function of the expansin gene *GsEXPB1* using transgenic soybean hairy roots and transgenic soybean lines.

### 3.1. Function and Mechanism of the GsEXPB1 Gene in Regulating Soybean Root Growth

Expansin is usually localized in the cell wall of plants, but there are also reports that it is localized on the cell membrane, for example HvEXPB7 in barley (*Hordeum vulgare*) [36] and OsEXPA17 in rice (*Oryza sativa*) [37]. In our previous work, we confirmed that the GsEXPB1 protein is localized in the cell wall [34]. The transcription of expansin genes, such as *BdEXPA27* in *Brachypodium distachyon* [38], *OsEXPB5* in rice [39], and *HvEXPB1* in barley [40], all of which are specifically transcribed in the roots, exhibits certain organ specificity. In a preliminary work, the transcription patterns of all expansin genes in the vegetative organs of wild soybean W05 were obtained through transcriptome sequencing analysis, and the *GsEXPB1* gene was identified as a root-specific transcription gene. Therefore, in this study, the function of the *GsEXPB1* gene in promoting soybean root growth was first identified through genetic transformation experiments of soybean hairy roots. The experimental results clearly showed that overexpression of the *GsEXPB1* significantly promoted the growth of soybean hairy roots, with significant relative increases in hairy root number, total root length, and total root weight compared to those of the wild type. Conversely, soybean hairy roots with RNAi silencing of the *GmEXPB4* gene (the homologous gene of *GsEXPB1* in soybean) exhibited the opposite phenotype, with significant growth inhibition, demonstrating the ability of *GsEXPB1* to promote soybean root growth (Figure 2). The function of expansin in promoting plant root growth has been widely confirmed by numerous studies. The first root-specifically expressed expansin, *GmEXP1*, discovered in soybean, has been proven to play a crucial role in regulating root development, particularly in the elongation of the main root and the initiation of lateral roots [41]. Similar results were also obtained in the transformation experiment of hairy roots in this study. To further analyze the function of *GsEXPB1* in promoting soybean root growth, soybean lines overexpressing *GsEXPB1* were also obtained in this study, and it was similarly found that the transgenic lines exhibited significantly superior root growth during the seedling stage compared to the wild type (under salt stress) (Figure 5 and Figure 6).

Regarding the mechanism of action of expansin, there are usually two hypotheses: the “acid growth” hypothesis and the “non-enzymatic mechanism” hypothesis. In the “acid growth” model, in many cases, the dependence of cell growth and cell wall extensibility on environmental pH increases significantly. A typical example is the auxin-mediated cell elongation process. Auxin activates proton pumps on the cell membrane, actively transports H^+^ to the cell wall, reduces pH to acidify the cell wall, and then activates the activity of expansin, promoting cell wall relaxation, leading to cell enlargement and facilitating water absorption. This phenomenon is common in rapidly growing organs, where the cells undergo several rounds of enlargement and typically contain water-filled vacuoles during this process [42]. In this study, both transgenic hairy roots and the roots of transgenic plants exhibited characteristics of rapid growth and high water content. It is speculated that when the GsEXPB1 protein is overexpressed, it is highly likely to further promote the relaxation of the cell wall in the roots. Although the plant cell wall plays an indispensable role as a key structure that maintains the stability of cell shape and limits cell size, excessive relaxation of the cell wall may significantly increase the absorption of water by the protoplasts (vacuoles) from the environment. This, in turn, can lead to the expansion of cell volume, resulting in the observed growth of the roots from a macroscopic perspective. This may be one of the potential reasons why the GsEXPB1 protein can promote root growth. Based on the current data, it is not clear whether the function of the *GsEXPB1* gene affects the number of cells in the roots of transgenic plants. It is speculated that the reason GsEXPB1 protein promotes root growth is related to auxin. This auxin-mediated “acidic growth” may be maintained by the accumulation of more auxin, which may originate from cotyledons (hairy roots) or actively growing meristems such as shoot apical meristems/root apical meristems (transgenic soybeans). Similarly, an increase in auxin content induces the meristematic zone of the root tip to produce more cells, thereby promoting the growth of transgenic roots. In this study, dynamic monitoring of auxin accumulation levels in the roots (root tips) of transgenic plants was not conducted. In subsequent research, relevant experiments will be carried out to further verify the aforementioned hypothesis. In another study conducted by our research team, overexpression of the *GsEXLB14* gene was found to alter the transcription levels of genes related to the MAPK cascade pathway, plant hormone signal transduction, and secondary metabolism processes [33]; among these, some key genes may be potential factors promoting root growth. However, the molecular mechanism by which *GsEXPB1* regulates soybean root growth remains unclear and requires further experimental confirmation.

### 3.2. Function and Main Pathways of GsEXPB1 Gene Regulating Soybean Salt Stress Tolerance

Compared to crops such as wheat, rice, and upland cotton (*Gossypium hirsutum*), soybeans are generally classified as salt-sensitive crops, and their yield and quality can significantly decrease under salt stress. With the increasing severity of soil salinization in farming worldwide and the continuous expansion of the area of salinized soil, cultivating soybean varieties with salt tolerance characteristics has crucial value and far-reaching significance for ensuring soybean yield, improving soybean quality, and maintaining sustainable agricultural development. Most research results indicate that expansin also plays an active role in regulating plant salt tolerance. For example, *NtEXPA4* in tobacco, *TaEXPA2* in wheat, *RhEXPA4* in *Rosa rugosa*, and *OsEXPA7* in rice can all enhance the salt tolerance of transgenic plants [36,39,42,43]. These genes exert their effects by increasing cell wall extensibility, reducing water loss, enhancing the activity of antioxidant enzyme systems, increasing the content of osmotic adjustment substances, and regulating Na^+^/K^+^ ion accumulation. In this study, to evaluate the function of *GsEXPB1* in enhancing the salt stress tolerance of soybean, relevant experiments were conducted using transgenic soybean hairy roots and soybean lines. In the hairy root experiment, compared to the wild type, overexpression of *GsEXPB1* significantly promoted the growth of soybean hairy roots under salt stress, while the growth of the RNAi group was significantly inhibited (Figure 2). Similarly, the soybean lines overexpressing *GsEXPB1* exhibited significantly superior phenotypes to the wild type under NaCl stress, both in terms of seed germination rate and seedling growth (Figure 5), as well as plant height, stem diameter, and root growth in different growth stages under saline soil cultivation conditions (Figure 6, Figure 11 and Figure 12). The aforementioned data demonstrate that the *GsEXPB1* gene possesses the ability to enhance soybean resistance to salt stress. Among various plant organs, the roots are the primary organs that respond to this stress. A well-developed root system aids plants in absorbing water and nutrients, thereby improving resistance to environmental stress [44]. Preliminary research data from our team show that under salt stress, the transcription of *GsEXPB1* in wild soybean roots exhibits a significant upregulation trend. This is generally considered an adaptive response, enabling roots to continue growing despite reduced cell turgor pressure, achieving an adaptive effect of increasing the root–shoot ratio, which allows roots to continue seeking water under salt stress conditions. Overexpression of *GsEXPB1* promotes the growth of transgenic soybean roots, which can alleviate the limitations imposed by salt stress on plant growth. This may be a potential reason why transgenic lines exhibit superior growth performance under salt stress compared to wild-type plants.

Under salt stress conditions, plants are usually subjected to three types of damage: oxidative stress, osmotic stress, and ionic stress. Low water potential disrupts the homeostasis of Na^+^/K^+^ ions in the cytoplasm, inducing toxic ion effects and subsequently leading to oxidative damage. Expansin has a good effect on alleviating these damage types in plants [27]. To further analyze the physiological mechanism of *GsEXPB1* regulating salt stress tolerance in soybeans, this study tested the antioxidant system, Na^+^/K^+^ ion content, and osmotic adjustment substance levels in transgenic soybean hairy roots and soybean strains. The results showed that under NaCl stress in soybean hairy roots, compared with the wild type, the activities of antioxidant enzymes (SOD, POD, CAT) and the content of reduced/oxidized glutathione were significantly increased in transgenic hairy roots, while the Na^+^ content decreased, and the K^+^ content increased, maintaining a lower Na^+^/K^+^ ratio. However, the RNAi roots exhibited the opposite trend (Figure 3). Under saline soil cultivation conditions, the observed and measured results in transgenic soybean lines were similar to the trends in hairy roots, and the results of reactive oxygen species and Evans blue staining also confirmed the accuracy of the data (Figure 8, Figure 9 and Figure 10). Regarding the accumulation levels of Na^+^ and K^+^ in plants under salt stress, we only conducted tests using hairy roots and soybean roots as materials. The relevant functions and pathways of *GsEXPB1* in the transport and accumulation of Na^+^ and K^+^ in the aboveground parts of plants are still unclear. Relevant experiments will be conducted in subsequent research. The physiological mechanism by which *GsEXPB1* enhances soybean salt tolerance is similar to that reported in previous studies. For example, in rice, the expansin gene *OsEXPA7* can improve the tolerance of rice plants to salt stress by synergistically regulating sodium ion transport processes, promoting effective removal of reactive oxygen species, and simultaneously facilitating moderate loosening of the cell wall [45]. Tobacco overexpressing the expansin gene *SmEXPA13* can reduce the relative conductivity and malondialdehyde content of plants under salt stress and improve salt tolerance by maintaining the Na^+^/K^+^ balance [29]. The *GsEXPB1* gene may also enhance the survival adaptability of soybeans under salt stress through similar multifaceted, synergistic effects.

In the research on the molecular mechanism of expansin regulating plant salt stress tolerance, the wild peanut (*Arachis*) expansin gene *AdEXLB8* was found to enhance tobacco’s tolerance to drought stress by activating the JA and ABA signaling pathways to regulate its antioxidant defense system [46]. The *Osmanthus fragrans* expansin gene *OfEXLA1* can promote the growth of *Arabidopsis thaliana* and enhance its salt tolerance and drought tolerance. Under salt and drought stress conditions, differential genes *OfABL4* and *OfABL5* were identified from the transcriptome. Further research found that OfABL4 and OfABL5 may bind to the *OfEXLA1* promoter to accumulate *OfEXLA1*, which improves the salt tolerance and drought tolerance of *Osmanthus fragrans* by responding to ABA signaling [47]. In another study conducted by our team, it was found that the overexpression of the expansin gene *GsEXLB14* from wild soybean significantly promoted the growth of transgenic soybean hairy roots under NaCl stress culture conditions. In these hairy roots, the transcription of genes encoding anion channel proteins, tonoplast ion pumps, and H^+^-ATPases, which are closely related to ion transport, was significantly upregulated. These genes play a crucial role in mitigating the ion toxicity damage caused by salt stress [33]. The molecular mechanism of *GsEXPB1* regulating salt stress tolerance in soybean lines has not been systematically studied, and this is also an important direction for elucidating the functional execution of this gene. In subsequent experiments, transcriptome sequencing analysis and other methods will be used for analysis.

### 3.3. The Application Value of the GsEXPB1 Gene in Soybean Breeding for Salt Stress Tolerance

Overexpression of expansin typically exerts positive effects on plant growth and development, as well as on environmental stress tolerance, thus demonstrating promising applications in crop breeding. For instance, tobacco plants overexpressing the expansin gene *NtEXPA11* exhibit enhanced growth phenotypes under normal growth conditions, with significantly increased biomass in roots and leaves compared to wild-type plants. Moreover, they are capable of developing significantly larger leaves and more lateral roots under salt stress, while maintaining normal growth patterns [48]. The lines overexpressing the soybean expansin gene *GmEXPA7* in hairy roots exhibited enhanced root structural characteristics, including significantly increased root length and root biomass, both under control and phosphorus-deficient conditions [49]. However, some research results indicate that overexpressing the expansin gene has no significant effect on the phenotype of transgenic lines under normal growth conditions, but it exhibits a function in enhancing tolerance under stress treatment. For example, cotton plants overexpressing the expansin gene *GhEXLB2* showed stronger drought resistance during germination, seedling, and flowering stages, but there were no significant differences in germination rate, root length, or hypocotyl length between the transgenic lines and the wild type under normal cultivation conditions [50]. Similarly, overexpressing the wild *Arachis* expansin gene *AdEXLB8* did not cause significant phenotypic changes in transgenic tobacco lines, but it enhanced the plants’ tolerance to drought stress [46]. Therefore, to elaborate on the function of the expansin gene, phenotypic or physiological data of transgenic plants under normal culture conditions and stress conditions are necessary. In this study, regarding the effect of overexpressing the *GsEXPB1* gene on the salt stress tolerance of cultivated soybeans, we only obtained relevant data under salt stress. In subsequent research, we will carry out experiments such as phenotypic monitoring of transgenic lines under normal growth conditions to enrich experimental data and provide more references for the application of this gene in molecular breeding of cultivated soybeans.

The germination rate of seeds is one of the key indicators for evaluating seed quality, as it directly affects the quality and yield of crops. Under salt stress, seed germination faces severe challenges. High-salt conditions can lead to an increase in osmotic pressure in soil solution, hindering the absorption of water by seeds, and ion toxicity can also damage the cellular structure and function of seeds. Expansin is involved in regulating the germination process of seeds. In the early stage of seed germination, the transcription levels of some key metabolic enzyme genes change, inducing the expression of expansin genes [51], which has been confirmed in many studies. For example, Montechiarini et al. found that abscisic acid downregulates the transcription of *GmEXP1* in soybeans, thereby inhibiting seed germination [52]. In wheat, the *TaEXPA6* gene has a high basal expression level during the early stages of seed development, and this may be one of the key genes involved in wheat grain type formation [53]. The expansin gene *DfEXPA1* in *Datura ferox* is expressed in both the endosperm and micropyle of the seed, and its expression is induced by gibberellin and phytochrome, playing an important role in breaking seed dormancy [54,55]. In this study, we found that soybean seeds overexpressing the *GsEXPB1* gene exhibited significantly higher germination rates under NaCl-simulated salt stress compared to wild-type seeds (Figure 5). For soybean cultivation in saline soil, the low germination rate has always been a limiting factor, so a higher germination rate is crucial for increasing soybean yield.

In addition to promoting plant cell wall relaxation to regulate plant growth, expansin also participates in regulating the component content of the cell wall. In *Arabidopsis thaliana* plants overexpressing the winter wheat expansin gene *TaEXPB7-B*, the contents of cellulose and lignin were significantly increased compared to the wild type [22]. In this study, the contents of cellulose, hemicellulose, and lignin were significantly increased in the roots of soybeans overexpressing the *GsEXPB1* gene (Figure 7), which may have a positive effect on enhancing the structure of plant roots and also has potential application value in the molecular breeding of soybeans.

Not only does expansin regulate the vegetative growth of plants, but some expansin genes have also been shown to perform important functions during the reproductive growth of plants. For example, in *Mirabilis jalapa*, the expansin gene is involved in the growth, blooming, and decay of flowers [56]. In this study, we found that overexpression of the expansin gene *GsEXPB1* significantly increased the number of flowers in transgenic soybeans (Figure 11E). The number of flowers can serve as an important indicator of crop yield and quality, indicating that the wild soybean *GsEXPB1* gene has the potential to enhance the reproductive capacity and environmental adaptability of soybeans. In terms of regulating soybean seed setting, the seed size of the overexpressing lines was significantly larger than that of the wild type, which in turn increased the 100-seed weight. However, due to the lack of a significant difference or significant reduction in the number of pods per plant and the number of seeds per plant compared to the wild type, the final yield per plant did not significantly increase compared to the wild type (Figure 12). Since this study featured a pot experiment with limited soil nutrient supply, the overexpression of the *GsEXPB1* gene significantly promoted the nutritional growth of soybeans under salt stress, which may have limited the reproductive growth process. Laboratory pot experiments cannot represent the true yield data. Therefore, to follow up, we will conduct field experiments in saline farmland to systematically monitor the yield indicators of transgenic lines. Meanwhile, the soybean material selected in this study is Williams 82, which is a traditional variety used in soybean genetic transformation research, rather than a commercial variety currently planted on a large scale. In subsequent research, we will use commercial varieties as materials to re-conduct molecular breeding work related to the *GsEXPB1* gene, with the aim of obtaining new soybean varieties with better salt stress tolerance. Based on the current experimental data, the wild soybean *GsEXPB1* gene has a good function and effect in improving soybean salt tolerance and still has high application value in molecular breeding work related to soybean salt tolerance.

## 4. Materials and Methods

### 4.1. Acquisition of Soybean Hairy Roots Through Overexpression of GsEXPB1 Gene and RNAi Silencing of GmEXPB4 Gene

The *GsEXPB1* gene was overexpressed using CaMV35S promoter. Using the pEASY-T3-GsEXPB1 cloning vector as a template, *Xba*I and *Sma*I restriction sites were added up- and downstream of the *GsEXPB1* gene, respectively (with the downstream stop codon removed), through PCR. The PCR product was purified and then digested with *Xba*I and *Sma*I restriction enzymes. Meanwhile, the pBI121-GUS expression vector was digested with *Xba*I and *Sma*I restriction endonucleases, and the digestion products were ligated using T4 DNA ligase to obtain the recombinant vector named pBI121-GsEXPB1-GUS. This vector was used to overexpress the GsEXPB1-GUS fusion protein (Figure 1A). Through homology Blast alignment with members of the expansin family in soybeans and amino acid phylogenetic tree analysis (Figure S1), it was determined that the homologous protein of GsEXPB1 in soybeans is GmEXPB4. The identity of the nucleotide sequence and amino acid sequence between the two genes is 99.88 and 99.64%, respectively. Based on the CDS sequence of the *GmEXPB4* gene, a specific fragment on this sequence was selected as the target sequence for RNAi silencing (Table S1). Using the pEASY-T3-GmEXPB4 cloning vector as a template, *Nco*I and *Swa*I restriction sites were added up- and downstream, respectively, of the forward target sequence, and *Sma*I and *Xba*I restriction sites were added up- and downstream, respectively, of the reverse target sequence through PCR. The purified PCR products were digested with the respective enzymes and ligated with the RNAi vector pFGC5941 using T4 DNA ligase to obtain the recombinant expression vector named pFGC4941-GmEXPB4. This vector was used for RNAi silencing of the *GmEXPB4* gene in soybeans (Figure 1B). PCR was performed using the EasyTaq^®^ DNA polymerase kit (TransGen Biotechnology Co., Ltd., Beijing, China), while the restriction endonucleases and T4 DNA ligase were provided by New England Biolabs (Beijing, China) Ltd.

The expression vectors pBI121-GsEXPB1-GUS and pFGC4941-GmEXPB4 were transformed into *Agrobacterium rhizogenes* K599 (Weidi Biotechnology Co., Ltd., Shanghai, China) using the freeze–thaw method. The transformation of soybean hairy roots was carried out according to Li et al. [57]. Soybean hairy roots induced by the K599 empty strain served as the control group, and soybean seeds were sourced from the variety Heilong 48 (provided by the College of Life Sciences of Northeast Agricultural University). The transcription and protein expression of the *GsEXPB1* gene in soybean hairy roots were detected using RT-PCR and GUS activity staining. RT-PCR was conducted according to Wang et al. [33]. The GUS staining kit and GUS activity assay kit were provided by Real-Times (Beijing, China) Biotechnology Co., Ltd. The transcription of the *GmEXPB4* gene in hairy roots was detected using RT-PCR and qRT-PCR, respectively. The TRANSGEN Top Green qPCR SuperMix kit (TransGen Biotechnology Co., Ltd., Beijing, China) was used for qRT-PCR experiments, and the Soybean *Actin* gene (GenBank: LOC100798052) was used as an internal reference gene. The primer sequences used in this study are shown in Table S2.

### 4.2. Phenotype Observation of Hairy Roots

The transgenic soybean hairy roots, which tested positive and had similar initial growth states, and the hairy roots induced by an empty bacterium (control group) were transferred to 1/2 MS solid medium for cultivation, with 150 mM NaCl serving as the salt stress treatment. Photographs were taken on days 0 and 7 for observation, and the changes in the number, total length, and total weight of the hairy roots were measured and statistically analyzed.

### 4.3. Measurement of Physiological Indicators of Hairy Roots

Hairy roots were sampled on days 0, 1, 3, 5, and 7 after salt stress treatment. After quick-freezing in liquid nitrogen, they were stored at −80 °C. Reagent kits (Suzhou Grace Biotechnology Co., Ltd., Suzhou, China, https://www.geruisi-bio.com/, accessed on 11 June 2024) were used to detect the activities of superoxide dismutase (SOD), peroxidase (POD), catalase (CAT), and the contents of reduced glutathione (GSH) and oxidized glutathione (GSSG) in the hairy roots. The reagent kit models used in the aforementioned experiments were as follows: superoxide dismutase (G0104F), peroxidase (G0108W), catalase (G0105W), reduced glutathione (G0206W48), and oxidized glutathione (G0207F). The content of Na^+^ and K^+^ in hairy roots was determined by atomic absorption spectrometry, with the service provided by Suzhou Michy Biomedical Technology Co., Ltd., Suzhou, China (https://jszmxswt6.etlong.com/, accessed on 20 June 2024).

### 4.4. Acquisition of GsEXPB1-Overexpressing Cultivated Soybean Lines

The *GsEXPB1* gene was overexpressed in cultivated soybeans using the CaMV35S promoter. *Asc*I and *Sac*I restriction sites were added up- and downstream of the CDS sequence of the *GsEXPB1* gene using PCR (with the downstream stop codon removed). The *GsEXPB1* gene was inserted into the pTF101 expression vector via double enzyme digestion, following the method described in Section 2.1. The obtained recombinant vector was named pTF101-GsEXPB1-HA (Figure 4A), and was used to overexpress the GsEXPB1-HA fusion protein. The pTF101-GsEXPB1-HA vector was transformed into *Agrobacterium tumefaciens* EHA105 using the freeze–thaw method. Genetic transformation of soybeans was conducted according to Gao et al. [58]. Soybean seeds of the Williams 82 variety (provided by the College of Life Sciences of Northeast Agricultural University) were used. RT-PCR and Western blot were employed to detect the transcription and protein expression, respectively, of the *GsEXPB1* gene in T1-generation soybean lines. For the RT-PCR experiment, the method was as described in Section 2.1. The expression of GsEXPB1 protein was detected using HA antibodies, with β-Tubulin protein serving as an internal control. T1-generation plants with positive identification results were cultured, and seeds were collected from each individual plant (T2 generation) for further experiments.

### 4.5. Analysis of Salt Tolerance of Transgenic Soybean Seeds

Williams 82 wild-type and T2-generation OE-GsEXPB1 soybean seeds of equal size and good appearance were selected as materials, and the following operations were performed in sequence: rinse with tap water for 15 min, treat with 75% ethanol for 1–2 min, and rinse with sterile water 2–3 times. The imbibed soybean seeds were picked up and spread evenly on a Petri dish (diameter, 9 cm) with two layers of filter paper inside. An amount of 5 mL of 150 mM NaCl solution was added as salt stress treatment, the Petri dish was placed at 25 °C for cultivation, and sterile water was added to replenish moisture in a timely manner. The germination rate was calculated based on the criterion that the radicle breaks through the seed coat by 0.5 cm. The germination test was concluded after 7 days. For morphological observation of seedlings, seeds were sown in germination bags after disinfection and cultured using Hoagland nutrient solution (25 °C, 16/8 h light cycle, 200 μmol/m^2^/s). An amount of 150 mM NaCl was used for salt stress treatment. Morphological observation and root length measurement were conducted on the 14th day after sowing.

### 4.6. Analysis of Salt Tolerance in Transgenic Soybean Plants

The salt tolerance of soybean lines overexpressing the *GsEXPB1* gene was tested using natural saline soil (provided by the Daqing Branch of the Heilongjiang Academy of Agricultural Sciences) as the cultivation medium. The soluble salt content in the soil was 0.28%, classified as moderately saline soil. Wild-type and T2 transgenic soybean seeds were sown in pots with a diameter of 27 cm for outdoor cultivation in May 2024, with timely water replenishment. On the 35th day of soybean planting, soybean seedlings were collected for phenotypic observation and physiological index determination. Plant height, stem diameter, root length, root number (total number per plant), root weight (fresh weight per plant), and nodule number (total number per plant) were measured. The content of cell wall components (cellulose, hemicellulose, lignin) in the roots was determined using reagent kits (Suzhou Grace Biotechnology Co., Ltd., Suzhou, China, https://www.geruisi-bio.com/, accessed on 12 August 2024). The reagent kit models used for the determination of cellulose, hemicellulose, and lignin content were G0715F, G0716W48, and G0708F, respectively. 3,3′-Diaminobenzidine (DAB), nitroblue tetrazolium (NBT), and Evans blue solution were used to detect ROS accumulation and the degree of cell damage in soybean leaves. The content of reactive oxygen species (ROS) in soybean leaves, including superoxide anion (O_2_^−^), hydrogen peroxide (H_2_O_2_), and malondialdehyde (MDA), was detected using reagent kits (Suzhou Grace Biotechnology Co., Ltd., Suzhou, China, https://www.geruisi-bio.com/, accessed on 16 August 2024). The activities of antioxidant enzymes, including superoxide dismutase (SOD), peroxidase (POD), and catalase (CAT), were also measured. Additionally, the accumulation of osmotic adjustment substances, including soluble sugar, soluble protein, and proline (Pro), as well as the chlorophyll content were assessed. The reagent kit models used in the aforementioned experiments were as follows: superoxide anion (G0116W), hydrogen peroxide (G0112F), malondialdehyde (G0110F), superoxide dismutase (G0104F), peroxidase (G0108W), catalase (G0105W), soluble sugar (G0501F), soluble protein (G0417W), proline (G0111W), and chlorophyll (G0601W). The content of Na^+^ and K^+^ in soybean roots was determined by atomic absorption spectrometry, with the service provided by Suzhou Michy Biomedical Technology Co., Ltd., Suzhou, China (https://jszmxswt6.etlong.com/, accessed on 19 August 2024). During the flowering period (68–92 days after planting), the plant height, stem diameter, and leaf count of soybean plants were measured. The number of flowers per plant was counted from the first day of flowering and was recorded every 4 days until the end of the soybean flowering period. In the full maturity stage of soybeans (159 days after planting), measurements of plant height, stem diameter, pod number per plant, seed setting quantity (number of seeds and weight), seed size, and 100-seed weight were taken.

### 4.7. Data Statistics

All experiments were repeated at least three times. Data were analyzed using Microsoft Office Excel 2021 software, and GraphPad Prism 9.0 software was used for plotting and significance analysis. Data are presented as mean ± standard deviation (Mean ± SD).

## 5. Conclusions

In this study, we utilized transgenic soybean hairy roots and soybean lines as materials to identify the regulatory capacity and physiological mechanisms for salt stress tolerance of the wild soybean *GsEXPB1* gene in cultivated soybean. The *GsEXPB1* gene significantly promotes the growth of soybean hairy roots under salt stress and enhances the ability to regulate antioxidant and sodium–potassium ion content. Additionally, overexpression of the *GsEXPB1* gene significantly increases the germination rate of soybean seeds under salt stress and promotes root growth. By actively regulating the antioxidant system, osmotic regulation system, chlorophyll content, cell wall components, and Na^+^/K^+^ levels, the nutritional growth of plants under saline soil cultivation conditions is enhanced, and the number of flowers, grain size, and 100-seed weight are increased. The above research results indicate that the GsEXPB1 gene derived from wild soybean has good application value in improving the salt stress tolerance of cultivated soybean and provides an experimental data reference for the application of wild soybean expansin gene resources in molecular breeding.

## Data Availability

Data are contained within the article and Supplementary Materials.

## Supplementary Materials

The following supporting information can be downloaded at https://www.mdpi.com/article/10.3390/plants14182851/s1: Figure S1: Amino acid phylogenetic tree of GsEXPB1 and soybean expansin family members; Table S1: Target sequence of *GmEXPB4* for RNAi silencing; Table S2: Primers used in this research.

## References

[1] Liu S., Zhang M., Feng F., Tian Z. (2020). Toward a “Green Revolution” for Soybean. Mol. Plant.

[2] Kofsky J., Zhang H., Song B.H. (2018). The Untapped Genetic Reservoir: The Past, Current, and Future Applications of the Wild Soybean (*Glycine soja*). Front. Plant Sci..

[3] Balasubramaniam T., Shen G., Esmaeili N., Zhang H. (2023). Plants’ Response Mechanisms to Salinity Stress. Plants.

[4] Sedivy E.J., Wu F., Hanzawa Y. (2017). Soybean domestication: The origin, genetic architecture and molecular bases. New Phytol..

[5] Zhao Y., Wang G., Zhao M., Wang M., Jiang M. (2022). Direct and indirect effects of soil salinization on soil seed banks in salinizing wetlands in the Songnen Plain, China. Sci. Total Environ..

[6] Meng F., Feng N., Zheng D., Liu M., Zhou H., Zhang R., Huang X., Huang A. (2024). Exogenous Hemin enhances the antioxidant defense system of rice by regulating the AsA-GSH cycle under NaCl stress. PeerJ.

[7] Liu L., Wang B. (2021). Protection of Halophytes and Their Uses for Cultivation of Saline-Alkali Soil in China. Biology.

[8] You H., Liu Y., Minh T.N., Lu H., Zhang P., Li W., Xiao J., Ding X., Li Q. (2020). Genome-wide identification and expression analyses of nitrate transporter family genes in wild soybean (*Glycine soja*). J. Appl. Genet..

[9] Hyten D.L., Song Q., Zhu Y., Choi I.Y., Nelson R.L., Costa J.M., Specht J.E., Shoemaker R.C., Cregan P.B. (2006). Impacts of genetic bottlenecks on soybean genome diversity. Proc. Natl. Acad. Sci. USA.

[10] Jiao Y., Bai Z., Xu J., Zhao M., Khan Y., Hu Y., Shi L. (2018). Metabolomics and its physiological regulation process reveal the salt-tolerant mechanism in Glycine soja seedling roots. Plant Physiol. Biochem..

[11] Zhang D.Y., Kumar M., Xu L., Wan Q., Huang Y.H., Xu Z.L., He X.L., Ma J.B., Pandey G.K., Shao H.B. (2017). Genome-wide identification of Major Intrinsic Proteins in *Glycine soja* and characterization of GmTIP2;1 function under salt and water stress. Sci. Rep..

[12] Feng X., Feng P., Yu H., Yu X., Sun Q., Liu S., Minh T.N., Chen J., Wang D., Zhang Q. (2020). GsSnRK1 interplays with transcription factor GsERF7 from wild soybean to regulate soybean stress resistance. Plant Cell Environ..

[13] Liu Y., Du H., Li P., Shen Y., Peng H., Liu S., Zhou G.A., Zhang H., Liu Z., Shi M. (2020). Pan-Genome of Wild and Cultivated Soybeans. Cell.

[14] Zheng H., Hou L., Xie J., Cao F., Wei R., Yang M., Qi Z., Zhu R., Zhang Z., Xin D. (2022). Construction of Chromosome Segment Substitution Lines and Inheritance of Seed-Pod Characteristics in Wild Soybean. Front. Plant Sci..

[15] McQueen-Mason S., Durachko D.M., Cosgrove D.J. (1992). Two endogenous proteins that induce cell wall extension in plants. Plant Cell..

[16] Li M., Liu T., Cao R., Cao Q., Tong W., Song W. (2023). Evolution and Expression of the Expansin Genes in Emmer Wheat. Int. J. Mol. Sci..

[17] Sampedro J., Cosgrove D.J. (2005). The expansin superfamily. Genome Biol..

[18] Guimaraes L.A., Mota A.P.Z., Araujo A.C.G., de Alencar Figueiredo L.F., Pereira B.M., de Passos Saraiva M.A., Silva R.B., Danchin E.G.J., Guimaraes P.M., Brasileiro A.C.M. (2017). Genome-wide analysis of expansin superfamily in wild *Arachis* discloses a stress-responsive expansin-like B gene. Plant Mol. Biol..

[19] Chen F., Bradford K.J. (2000). Expression of an expansin is associated with endosperm weakening during tomato seed germination. Plant Physiol..

[20] Liu W., Xu L., Lin H., Cao J. (2021). Two Expansin Genes, *AtEXPA4* and *AtEXPB5*, Are Redundantly Required for Pollen Tube Growth and *AtEXPA4* Is Involved in Primary Root Elongation in *Arabidopsis thaliana*. Genes.

[21] Fan N., Xu Q., Yang Z., Zhuang L., Yu J., Huang B. (2023). Identification of expansin genes as promoting or repressing factors for leaf elongation in tall fescue. Physiol. Plant..

[22] Feng X., Xu Y., Peng L., Yu X., Zhao Q., Feng S., Zhao Z., Li F., Hu B. (2019). *TaEXPB7-B*, a β-expansin gene involved in low-temperature stress and abscisic acid responses, promotes growth and cold resistance in *Arabidopsis thaliana*. J. Plant Physiol..

[23] Han Z., Liu Y., Deng X., Liu D., Liu Y., Hu Y., Yan Y. (2019). Genome-wide identification and expression analysis of expansin gene family in common wheat (*Triticum aestivum* L.). BMC Genom..

[24] Bernal-Gallardo J.J., González-Aguilera K.L., de Folter S. (2024). EXPANSIN15 is involved in flower and fruit development in *Arabidopsis*. Plant Reprod..

[25] Dong C., Zou X., Gao Q.H. (2022). Genome-wide identification of expansin in Fragaria vesca and expression profiling analysis of the FvEXPs in different fruit development. Gene.

[26] Narváez-Barragán D.A., Tovar-Herrera O.E., Segovia L., Serrano M., Martinez-Anaya C. (2020). Expansin-related proteins: Biology, microbe-plant interactions and associated plant-defense responses. Microbiology.

[27] Van Zelm E., Zhang Y., Testerink C. (2020). Salt Tolerance Mechanisms of Plants. Annu. Rev. Plant Biol..

[28] Zörb C., Mühling K.H., Kutschera U., Geilfus C.M. (2015). Salinity stiffens the epidermal cell walls of salt-stressed maize leaves: Is the epidermis growth-restricting?. PLoS ONE.

[29] Zhang J., Wang L., Wu D., Zhao H., Gong L., Xu J. (2024). Regulation of SmEXPA13 expression by SmMYB1R1-L enhances salt tolerance in *Salix matsudana* Koidz. Int. J. Biol. Macromol..

[30] Chen Y., Han Y., Kong X., Kang H., Ren Y., Wang W. (2017). Ectopic expression of wheat expansin gene *TaEXPA2* improved the salt tolerance of transgenic tobacco by regulating Na^+^/K^+^ and antioxidant competence. Physiol. Plant..

[31] Kuluev B., Avalbaev A., Mikhaylova E., Nikonorov Y., Berezhneva Z., Chemeris A. (2016). Expression profiles and hormonal regulation of tobacco expansin genes and their involvement in abiotic stress response. J. Plant Physiol..

[32] Zhu Y., Wu N., Song W., Yin G., Qin Y., Yan Y., Hu Y. (2014). Soybean (*Glycine max*) expansin gene superfamily origins: Segmental and tandem duplication events followed by divergent selection among subfamilies. BMC Plant Biol..

[33] Wang L., Zhang T., Li C., Zhou C., Liu B., Wu Y., He F., Xu Y., Li F., Feng X. (2024). Overexpression of Wild Soybean Expansin Gene *GsEXLB14* Enhanced the Tolerance of Transgenic Soybean Hairy Roots to Salt and Drought Stresses. Plants.

[34] Feng X., Li C., He F., Xu Y., Li L., Wang X., Chen Q., Li F. (2022). Genome-Wide Identification of Expansin Genes in Wild Soybean (*Glycine soja*) and Functional Characterization of *Expansin B1* (*GsEXPB1*) in Soybean Hair Root. Int. J. Mol. Sci..

[35] Guo X., Jiang J., Liu Y., Yu L., Chang R., Guan R., Qiu L. (2021). Identification of a Novel Salt Tolerance-Related Locus in Wild Soybean (*Glycine soja* Sieb. & Zucc.). Front. Plant Sci..

[36] He X., Zeng J., Cao F., Ahmed I.M., Zhang G., Vincze E., Wu F. (2015). *HvEXPB7*, a novel β-expansin gene revealed by the root hair transcriptome of Tibetan wild barley, improves root hair growth under drought stress. J. Exp. Bot..

[37] ZhiMing Y., Bo K., XiaoWei H., ShaoLei L., YouHuang B., WoNa D., Ming C., Hyung-Taeg C., Ping W. (2011). Root hair-specific expansins modulate root hair elongation in rice. Plant J..

[38] Chen S., Luo Y., Wang G., Feng C., Li H. (2020). Genome-wide identification of expansin genes in *Brachypodium distachyon* and functional characterization of *BdEXPA27*. Plant Sci..

[39] Won S.K., Choi S.B., Kumari S., Cho M., Lee S.H., Cho H.T. (2010). Root hair-specific *EXPANSIN B* genes have been selected for Graminaceae root hairs. Mol. Cells.

[40] Kwasniewski M., Szarejko I. (2006). Molecular cloning and characterization of beta-expansin gene related to root hair formation in barley. Plant Physiol..

[41] Lee D.K., Ahn J.H., Song S.K., Choi Y.D., Lee J.S. (2003). Expression of an expansin gene is correlated with root elongation in soybean. Plant Physiol..

[42] Cosgrove D.J. (2021). Expanding wheat yields with expansin. New Phytol..

[43] Zhang W., Yan H., Chen W., Liu J., Jiang C., Jiang H., Zhu S., Cheng B. (2014). Genome-wide identification and characterization of maize expansin genes expressed in endosperm. Mol. Genet. Genom..

[44] Zou X., Liu L., Hu Z., Wang X., Zhu Y., Zhang J., Li X., Kang Z., Lin Y., Yin C. (2021). Salt-induced inhibition of rice seminal root growth is mediated by ethylene-jasmonate interaction. J. Exp. Bot..

[45] Jadamba C., Kang K., Paek N.C., Lee S.I., Yoo S.C. (2020). Overexpression of Rice *Expansin7* (*Osexpa7*) Confers Enhanced Tolerance to Salt Stress in Rice. Int. J. Mol. Sci..

[46] Brasileiro A.C.M., Lacorte C., Pereira B.M., Oliveira T.N., Ferreira D.S., Mota A.P.Z., Saraiva M.A.P., Araujo A.C.G., Silva L.P., Guimaraes P.M. (2021). Ectopic expression of an expansin-like B gene from wild Arachis enhances tolerance to both abiotic and biotic stresses. Plant J..

[47] Dong B., Wang Q., Zhou D., Wang Y., Miao Y., Zhong S. (2024). Abiotic stress treatment reveals expansin like A gene *OfEXLA1* improving salt and drought tolerance of *Osmanthus fragrans* by responding to abscisic acid. Hortic. Plant J..

[48] Marowa P., Ding A., Xu Z., Kong Y. (2020). Overexpression of *NtEXPA11* modulates plant growth and development and enhances stress tolerance in tobacco. Plant Physiol. Biochem..

[49] Liu X., Cai Y., Yao W., Chen L., Hou W. (2024). The soybean NUCLEAR FACTOR-Y C4 and α-EXPANSIN 7 module influences phosphorus uptake by regulating root morphology. Plant Physiol..

[50] Zhang B., Chang L., Sun W., Ullah A., Yang X. (2021). Overexpression of an expansin-like gene, *GhEXLB2* enhanced drought tolerance in cotton. Plant Physiol. Biochem..

[51] Wei P.C., Zhang X.Q., Zhao P., Wang X.C. (2011). Regulation of stomatal opening by the guard cell expansin AtEXPA1. Plant Signal Behav..

[52] Montechiarini N.H., Delgado L., Morandi E.N., Néstor J.C., Gosparini C.O. (2021). The expansin *EXP1* gene in the elongation zone is induced during soybean embryonic axis germination and differentially expressed in response to ABA and PEG treatments. Seed Sci. Res..

[53] Lizana X.C., Riegel R., Gomez L.D., Herrera J., Isla A., McQueen-Mason S.J., Calderini D.F. (2010). Expansins expression is associated with grain size dynamics in wheat (*Triticum aestivum* L.). J. Exp. Bot..

[54] Arana M.V., de Miguel L.C., Sánchez R.A. (2006). A phytochrome-dependent embryonic factor modulates gibberellin responses in the embryo and micropylar endosperm of *Datura ferox* seeds. Planta.

[55] Mella R.A., Burgin M.J., Sánchez R.A. (2004). Expansin gene expression in *Datura ferox* L. seeds is regulated by the low-fluence response, but not by the high-irradiance response of phytochromes. Seed Sci. Res..

[56] Meir S., Hunter D.A., Chen J.C., Halaly V., Reid M.S. (2006). Molecular changes occurring during acquisition of abscission competence following auxin depletion in *Mirabilis jalapa*. Plant Physiol..

[57] Li C., Zhang H., Wang X., Liao H. (2014). A comparison study of Agrobacterium-mediated transformation methods for root-specific promoter analysis in soybean. Plant Cell Rep..

[58] Gao L., Ding X., Li K., Liao W., Zhong Y., Ren R., Liu Z., Adhimoolam K., Zhi H. (2015). Characterization of Soybean mosaic virus resistance derived from inverted repeat-SMV-HC-Pro genes in multiple soybean cultivars. Theor. Appl. Genet..

