# Supplementary material for: Overexpression of the Wild Soybean Expansin Gene GsEXPB1 Enhances Salt Stress Tolerance in Transgenic Soybeans
Source: Plants (Basel). 2025 Sep 12;14(18):2851. doi: 10.3390/plants14182851 (PMC12473410; doi:10.3390/plants14182851)
Supplement: Supplementary file 1 [file plants-14-02851-s001.zip › plants-3811584-supplementary.pdf]

Figure S1

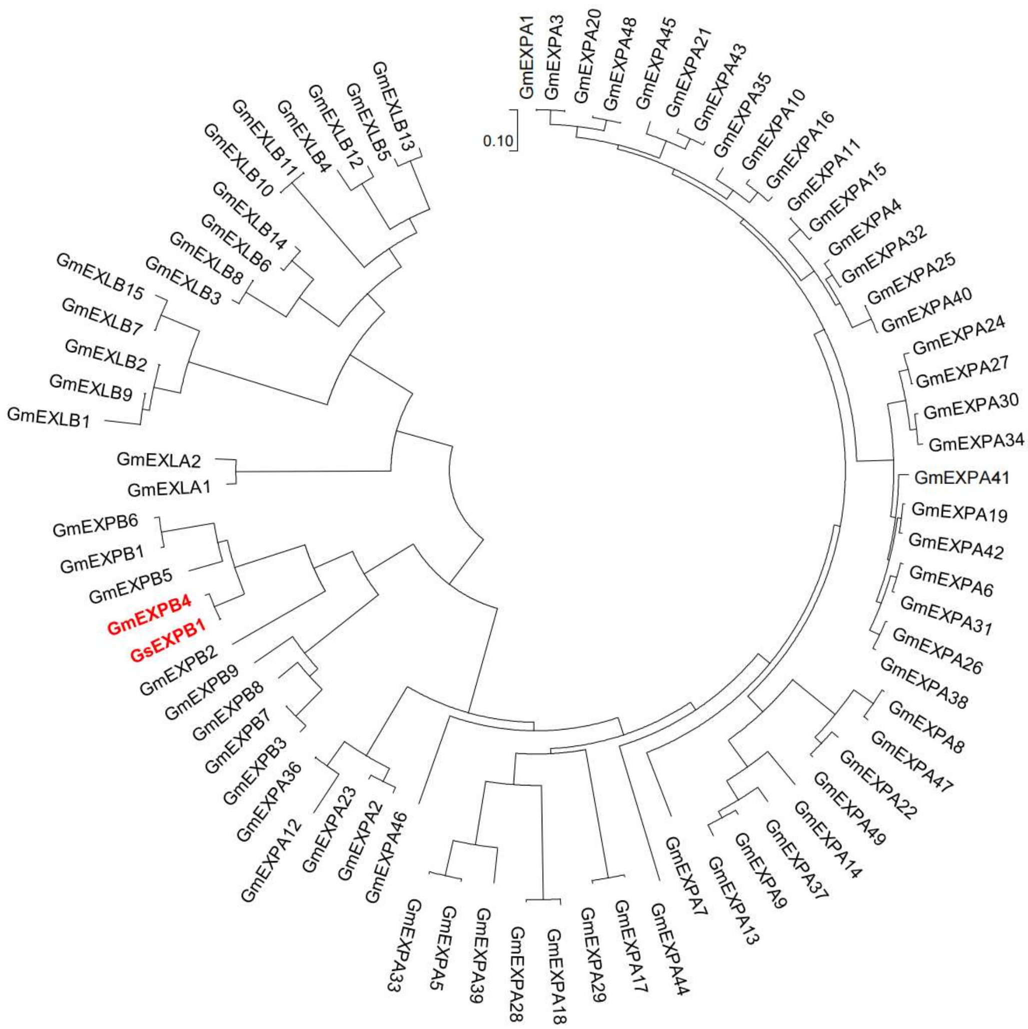

Figure S1. Amino acid phylogenetic tree of GsEXPB1 and soybean expansin family members.

Table S1

Table S1 Target sequence of *GmEXPB4* for RNAi silencing

Name	Sequence（5'-3'）
Positive target sequence	ATGGCTCCTACACTTCAACGTGCACTTTCTCATCTGCTCACTCTTGTAGCTTCACTTTCAAT<br>ACTCCTAGTGGTACCCTCCTCTTGTTTCAACCCTAAAAAGATTGTGAATGCTTCCTATGCT<br>TCATACTCCTTATATGGTTCAGAT

Table S2

Table S2 Primers used in this research

Name	Primer Sequences（5'-3'）	Purpose
Xba Ⅰ-*GsEXPB1*-F	GCTCTAGAATGGCTCCTACACTTC	Restriction site addition
Sma Ⅰ-*GsEXPB1*-R	TCCCCCGGGAAAATTGACAATTGAT	
Sma Ⅰ-Positive *GmEXPB4*-F	TCCCCCGGGATGGCTCCTACACTTC	Restriction site addition
Xba Ⅰ-Positive *GmEXPB4*-R	GCTCTAGAATCTAACCATAT	
Nco Ⅰ-Reverse *GmEXPB4*-F	GACCATGGAGATTGGTATA	
Swa Ⅰ-Reverse *GmEXPB4*-R	CGCGATTTAAATTACCGAGGATGTGAAG	
Actin-F	CGGTGGTTCTATCTTGGCATC	Soybean *Actin*
Actin-R	GTCTTTCGCTTCAATAACCCTA	
Y-*GmEXPB4*-F	GGTGTAGCCACTTGGTATGGAC	qRT-PCR
Y-*GmEXPB4*-R	GGGAGTGATGACTACCCTTACAGAA	
Asc Ⅰ-*GsEXPB1*-F	GCGGCGCGCCATGGCTCCTACACTTC	Restriction site addition
Sac Ⅰ-*GsEXPB1*-R	TCGAGCTCAAAATTGACAATTGAT	
